# Exploring the Impact of Music Preferences on Depressive Symptoms and Meaning in Life: A Network Analysis Approach

**DOI:** 10.3390/bs15101311

**Published:** 2025-09-25

**Authors:** Qizong Yue, Yuqi Lin, Bo Yang, Maoping Zheng

**Affiliations:** 1 School of Music, Southwest University, Chongqing 400700, China; yueqizong@outlook.com; 2 Faculty of Education, University of Macau, Taipa, Macau, China; yc37120@um.edu.mo; 3 School of Psychology, Southwest University, Chongqing 400700, China

**Keywords:** network analysis, music preference, depressive symptoms, meaning in life, mental health, sad music, happy music, music therapy

## Abstract

This study investigates how preferences for sad or happy music influence the network structures linking depressive symptoms and meaning in life. Analyzing data from 1681 college students, results indicate that individuals who listen to sad music display a denser network structure with stronger connections between depressive symptoms and meaning in life, while those favoring happy music exhibit a more dispersed network with weaker connections. The Sad Music Group showed higher global strength, suggesting a tightly knit network, whereas the Happy Music Group had lower global strength, implying greater flexibility among nodes. These findings highlight distinct network configurations between the two groups, offering insights into the interplay between music engagement and psychological well-being. By identifying key nodes and connectivity, we can develop more targeted therapy interventions. However, it is crucial to consider individual differences and contextual factors that influence how music affects psychological well-being.

## 1. Introduction

The impact of listening to music daily, whether sad or happy, on depressive moods and the sense of meaning in life is an intricate subject that has garnered interest within the fields of psychology and music therapy. Research indicates that daily engagement with different types of music can yield varied emotional responses and implications for mental health. Listening to sad music is often a topic of considerable debate due to its paradoxical nature. It is known that many individuals report deriving comfort and emotional relief from sad music, a phenomenon frequently linked to a process called emotional contagion, where listeners experience parallel emotions to those expressed in the music. This process can enhance feelings of empathy and communal understanding, potentially allowing listeners to feel less isolated during moments of sadness. In essence, sad music can provide a reflective space for individuals, helping them to process grief and melancholy, which may contribute to an enhanced sense of meaning by validating their emotional experiences and fostering a deeper connection with one’s inner self (Eerola et al., 2016).

Conversely, while listening to sad music can be therapeutic for some, it also poses risks for individuals with tendencies toward rumination or severe depression. Repeated engagement can intensify depressive symptoms and reinforce negative emotional cycles, leading to hopelessness and a reduced sense of purpose (Arens & Stangier, 2020; Eerola et al., 2016; Garrido et al., 2017). In contrast, happy music typically induces positive mood changes and promotes feelings of joy and excitement. Positive music listening is associated with various beneficial outcomes, including improved mood regulation, enhanced social connections, and increased motivation. In the context of depressive mood, listening to upbeat, happy music can act as a counterbalance to negative emotions, fostering a sense of light-heartedness that can encourage individuals to engage more fully with their surroundings and potentially discover new meanings in their daily lives (Blasco-Magraner et al., 2023; Palmiero et al., 2023). Happy music has also been linked to a broadened scope of attention, allowing individuals to engage more flexibly with their thoughts and environments, thus contributing positively to their overall well-being (Putkinen et al., 2017).

The relationship between music engagement, depression, and meaning in life may also be linked through the construct of meaningfulness. Studies have indicated that individuals who actively engage with meaningful music—whether it be upbeat genres that foster joy or poignant pieces that resonate with personal experiences—tend to report greater levels of life satisfaction and meaning (Eerola & Peltola, 2016; Saarikallio et al., 2015). The key to this relationship appears to be not just the type of music but also the listener’s psychological state and the context in which the music is consumed. For instance, the hedonic value gained from upbeat music may be more pronounced in individuals seeking joy and escape from their mundane routines, thereby providing an effective counterbalance to depressive tendencies. Network analysis has begun to illuminate these dynamics quantitatively, showcasing the interconnectedness of daily music listening habits, emotional responses, and mental health metrics. By analyzing daily music engagement patterns, researchers can identify whether an individual’s listening habits are more dominantly associated with depressive symptomatology or with enhanced feelings of purpose and meaning in life (Chao et al., 2020; Eerola & Peltola, 2016).

Network analysis has emerged as a powerful tool in psychological research, offering a novel perspective on the interrelationships between psychological constructs. This approach models psychological phenomena as networks of nodes (representing variables) connected by edges (indicating statistical relationships)—not to establish causality, but to map correlational patterns between constructs (Fried et al., 2016). Prior research has utilized network analysis to explore various psychological domains, yet limited attention has been given to how individual differences, such as music preferences, may shape these networks. Music is known to have a profound impact on emotions and cognition, with different genres potentially influencing psychological states in distinct ways, though this relationship may be modulated by unmeasured factors that are well documented in the literature but not assessed here. These include (1) music-related health perceptions (e.g., via the Healthy-Unhealthy Music Scale, which captures adaptive vs. maladaptive music engagement; Saarikallio et al., 2015); (2) dispositional rumination (a tendency linked to amplified negative effects of sad music; Garrido et al., 2017); and (3) empathy (a trait associated with emotional resonance to sad music and potential buffering of depressive symptoms; Eerola et al., 2016). Three experimental hypotheses guide this study, focusing on correlations between music preferences, behavioral dispositions (e.g., emotion processing tendencies), and psychological states:

**H1.**  *Preference for sad music (vs. happy music) will correlate with a tighter network structure (stronger connections) between depressive symptoms and meaning in life, reflecting a dispositional tendency toward emotional rumination.*

**H2.**  *Preference for happy music (vs. sad music) will correlate with a looser network structure (weaker connections) between depressive symptoms and meaning in life, reflecting a dispositional tendency toward positive emotion regulation (Putkinen et al., 2017).*

**H3.**  *Certain nodes related to meaning in life and depressive symptoms will exhibit higher centrality in the Sad Music Group compared to the Happy Music Group.*

## 2. Materials and Methods

### 2.1. Participants and Data Collection

The data used in this study were collected from a sample of participants who completed a set of questionnaires measuring meaning in life (via the Meaning in Life Questionnaire, MLQ), depressive symptoms (via the Patient Health Questionnaire, PHQ-9), and music preference. We provide group-level descriptive statistics (mean, standard deviation) for project labels (Life1-Life10), PHQ-9, and MLQ subscales (see Appendix A Table A1 and Table A2).

#### 2.1.1. Recruitment Procedure

Participants were recruited between March 2023 and June 2023 via a multi-site convenience sampling strategy across three public universities in Chongqing, China (Southwest University, Chongqing Normal University, and Chongqing University of Posts and Telecommunications). Recruitment channels included the following: (1) in-class announcements by research assistants (targeting undergraduate and postgraduate students across majors); (2) online postings on university official student forums and WeChat groups (with a link to the electronic questionnaire); and (3) on-campus poster advertisements (displayed in libraries, dormitory lobbies, and music/psychology department buildings). Prior to recruitment, all research assistants received training on standardized participant briefing (e.g., explaining the study’s purpose, voluntary participation, and data anonymity).

#### 2.1.2. Inclusion and Exclusion Criteria

Participants were selected based on predefined inclusion and exclusion criteria to ensure sample relevance and data quality.

Inclusion criteria required participants to (1) be currently enrolled as a full-time college student (undergraduate or postgraduate) at the recruited universities; (2) be aged 18–28 years (consistent with the typical college student age range in China); (3) be able to read and understand simplified Chinese (to complete the questionnaires); (4) report regular music listening habits (defined as listening to music at least 3 times per week for ≥30 min per session over the past month); (5) provide informed consent to participate.

Exclusion criteria included the following: (1) self-reported diagnosis of a severe mental illness (e.g., schizophrenia, bipolar disorder) or neurological disorder (e.g., epilepsy) via a screening item in the questionnaire; (2) current use of psychiatric medications (e.g., antidepressants, mood stabilizers) that may affect emotional states; (3) inability to complete the questionnaire independently (e.g., due to cognitive impairment); (4) duplicate submissions (identified via matching IP addresses and student ID numbers, where provided).

#### 2.1.3. Data Collection Method

Data were collected using a cross-sectional, self-administered electronic questionnaire (hosted on the Chinese online survey platform “WenJuanXing,” a widely used tool for academic research in China). The questionnaire included three sections: (1) a screening module (to assess eligibility via inclusion/exclusion criteria); (2) the core measurement scales (MLQ, PHQ-9, and music preference question); and (3) a demographic module (gender, age, major, grade). The dataset was divided into two groups based on this response: (1) Sad Music Group (SMG), participants who selected “sad music” as their most frequently listened to type, and (2) Happy Music Group (HMG), participants who selected “happy music” as their most frequently listened to type. Participants completed the questionnaire independently, with no time limit (average completion time: 12 ± 3 min). To ensure data quality, the questionnaire included attention check items (e.g., “Please select ‘strongly agree’ for this item”)—responses failing ≥1 attention check were excluded from analysis.

This study was conducted in accordance with the Declaration of Helsinki and formally approved by the Human Research Ethics Committee of the Department of Psychology at Southwest University (Approval No.: H24188; Date: 8 November 2024). Informed consent was obtained from all participants prior to questionnaire completion, with details on data storage (encrypted servers) and use (academic research only) provided in the consent form.

#### 2.1.4. Response Rates and Sample Size

A total of 1850 potential participants accessed the electronic questionnaire. After applying exclusion criteria, (1) 42 participants were excluded for failing attention checks; (2) 38 participants were excluded for not meeting the regular music listening habit criterion; (3) 65 participants were excluded for self-reporting severe mental/neurological disorders or psychiatric medication use; (4) 24 participants were excluded for duplicate submissions. This resulted in 1681 valid questionnaires, yielding an effective response rate of 90.9% (1681/1850). As previously reported, the final sample included 1121 male students (66.7%) and 560 female students (33.3%), with 320 in the Sad Music Group (19.0%) and 1361 in the Happy Music Group (81.0%).

### 2.2. Scales

#### 2.2.1. The Meaning in Life Questionnaire (MLQ)

The MLQ is a self-assessment scale consisting of 10 items, encompassing two dimensions, presence of meaning and search for meaning, with 5 items each. It employs a Likert 7-point scoring system (1 = “strongly disagree”; 7 = “strongly agree”). Among the 10 items of this scale, except for item 9 which is reverse-scored, all other items are positively scored. The presence of meaning (MLQ-P) includes items 1, 4, 5, 6, and 9; the search for meaning (MLQ-S) includes items 2, 3, 7, 8, and 10. The measurement indicator is the average score of each dimension. Higher scores indicate higher levels of meaning presence or meaning seeking (Steger et al., 2006). Life1: purpose-seeking; Life2: purpose absence; Life3: meaning exploration; Life4: meaning awareness; Life5: meaning stimulus seeking; Life6: purpose continuous exploration; Life7: direction clarity; Life8: meaning source awareness; Life9: purpose satisfaction; and Life10: importance pursuit.

#### 2.2.2. The Patient Health Questionnaire (PHQ-9)

The Patient Health Questionnaire (PHQ-9), a self-assessment tool for depression, was jointly developed by Kroenke et al. (2001). In this study, we employed the PHQ-9 to evaluate participants’ depressive symptoms. The PHQ-9 is a widely used self-rating scale designed for screening and assessing the severity of depressive symptoms. This scale consists of 9 items corresponding to the diagnostic criteria for depression, with each item scored from 0 (“not at all”) to 3 (“nearly every day”) based on symptom frequency. The total score ranges from 0 to 27, with higher scores indicating more severe depressive symptoms. According to the PHQ-9 scoring criteria, participants’ depressive symptoms were categorized into the following five levels: 0–4 points: no depressive symptoms; 5–9 points: mild depression; 10–14 points: moderate depression; 15–19 points: moderately severe depression; and 20–27 points severe depression. Due to its brevity and effectiveness, the PHQ-9 scale has been extensively utilized in clinical and research settings, providing robust support for the preliminary screening and assessment of depression. In this study, participants were instructed to rate each item based on their experiences over the past two weeks. All ratings were independently completed by participants at the outset of the study to ensure objectivity and reliability of the results. PHQ-9 symptoms: PHQ1: anhedonia; PHQ2: sad mood; PHQ3: sleep; PHQ4: fatigue; PHQ5: appetite; PHQ6: guilt; PHQ7: concentration; PHQ8: motor; and PHO9: suicide.

#### 2.2.3. Music Preference Classification

Music preference was assessed using a single-item measure designed to capture participants’ dominant listening habits over the past 30 days. Participants were asked “Over the past 30 days, which type of music have you listened to most frequently?” Response options included the following: (1) sad music (e.g., melancholic ballads, minor-key instrumental music, songs with sorrowful lyrics) and (2) happy music (e.g., upbeat pop, major-key folk music, songs with joyful lyrics). Based on their responses, participants were categorized into the Sad Music Group (SMG) or the Happy Music Group (HMG). This binary classification was used as the grouping variable in subsequent network analyses.

### 2.3. Network Construction

The data were imported into R (version 4.3.1) using the readxl package, and relevant variables (MLQ items, PHQ-9 items, music preference group) were extracted for network analysis. The network analysis was performed using the R programming language with several packages, including qgraph, bootnet, and NetworkComparisonTest. The steps involved in network construction were as follows:

Data Preparation: The variables measuring meaning in life and depressive symptoms were organized into separate data frames for each group. The variables were categorized into two groups: Life (meaning in life measures) and PHQ (depressive symptoms).

Missing data handling: Cases with >5% missing values (*n* = 12) were excluded; remaining missing values (<2%) were imputed using multiple imputation (*m* = 5) via the mice package in R. No nonparanormal transformation was applied, as polychoric correlations inherently accommodate ordinality without assuming normality.

Standardization: All items were z-scored (M = 0, SD = 1) to ensure comparable edge weights across variables with different response scales.

To address potential item redundancy, we conducted a goldbricker test using the goldbricker package, which identifies items that do not contribute unique variance. Results indicated no redundant items (all items had uniqueness values > 0.30, exceeding the recommended threshold of 0.20), confirming that each PHQ-9 and MLQ item captures distinct variance relevant to depressive symptoms or meaning in life.

Network Estimation: The Gaussian graphical model (GGM) was employed to estimate the network structures for both groups using the graphical LASSO method in combination with Extended Bayesian Information Criterion (EBIC) model selection (Epskamp et al., 2018; van Borkulo et al., 2014). This approach allowed for the estimation of partial correlation coefficients between variables while controlling for other variables in the dataset. The network models were estimated using the R-package qgraph version 1.9.8 (Epskamp et al., 2012).

Network Visualization: The qgraph package was used to visualize the estimated networks for both groups, with nodes representing the variables and edges representing the partial correlation coefficients. Different colors and layouts were applied to distinguish between the two groups and enhance the interpretability of the network structures.

### 2.4. Accuracy and Stability Assessment

To assess the accuracy and stability of the estimated networks, the following methods were employed:

Bootstrapping Procedures: Non-parametric bootstrapping was used to estimate confidence intervals (CIs) for edge weights and centrality indices. This involved resampling the data with replacement to create multiple bootstrap samples, estimating the network parameters for each sample, and calculating the CIs based on the distribution of the bootstrap estimates. The bootnet package facilitated this process, allowing for the estimation of bootstrapped CIs and the visualization of the results.

Correlation Stability Coefficient (CS-coefficient): This measure was used to quantify the stability of centrality indices under case-dropping subset bootstrap. It indicates the maximum proportion of cases that can be dropped while maintaining a certain correlation (default 0.7) between the original centrality indices and those obtained from the subsetted data. A higher CS-coefficient value suggests greater stability of the centrality indices.

Bridge Statistics: Bridge strength and bridge expected influence were calculated to assess the connectivity between different communities within the networks. This helped to identify potential bridge nodes that may play a significant role in connecting different aspects of meaning in life and depressive symptoms.

Effect Size Calculation: For statistically significant group differences in network metrics (e.g., global strength, node centrality), Cohen’s *d* was calculated as the effect size (Cohen, 1988), with thresholds: *d* < 0.2 (trivial), 0.2–0.5 (small), 0.5–0.8 (medium), and ≥0.8 (large). Descriptive statistics (mean ± SD) for PHQ-9 (depressive symptoms) and MLQ (meaning in life) were used to support effect size calculations.

Network Comparison and Effect Size Calculation: To test the influence of music preferences on the psychological well-being network (integrating depressive syndrome and life meaning experience), the NetworkComparisonTest package (version 2.2.1) was used to conduct three levels of invariance tests: (1) overall network structure invariance (test statistic M); (2) global strength invariance (test statistic S, measuring average connection strength between nodes); (3) node-level centrality invariance (via the centralityTest function with 1000 bootstraps to control Type I error).

For all statistically significant group differences, Cohen’s *d* was calculated as the primary effect size metric, with interpretation based on standard thresholds: *d* < 0.2 (trivial effect), 0.2 ≤ *d* < 0.5 (small effect), 0.5 ≤ *d* < 0.8 (medium effect), and *d* ≥ 0.8 (large effect). Descriptive statistics (mean ± standard deviation, M ± SD) for core variables, including depressive syndrome (PHQ-9 total score) and life meaning experience (MLQ-P: Presence of Meaning, MLQ-S: Search for Meaning), were also collected to support effect size calculation and result interpretation.

## 3. Results

### 3.1. Descriptive Statistical Results of Major Demographic Characteristics

A total of 1681 valid questionnaires were collected from different genders, age groups, and music preferences of college students. In terms of gender distribution, there were 1121 male students, accounting for 66.7% of the total sample, while 560 female students accounted for 33.3%. Regarding music preferences, 320 students indicated that the most frequently listened to type of music in the past month was sad music, making up 19.0% of the total sample, whereas 1361 students indicated that the most frequently listened to type of music was happy music, accounting for 81.0%.

### 3.2. Exploratory Network Analysis of Meaning in Life and Depressive Symptoms

**Figure 1:**
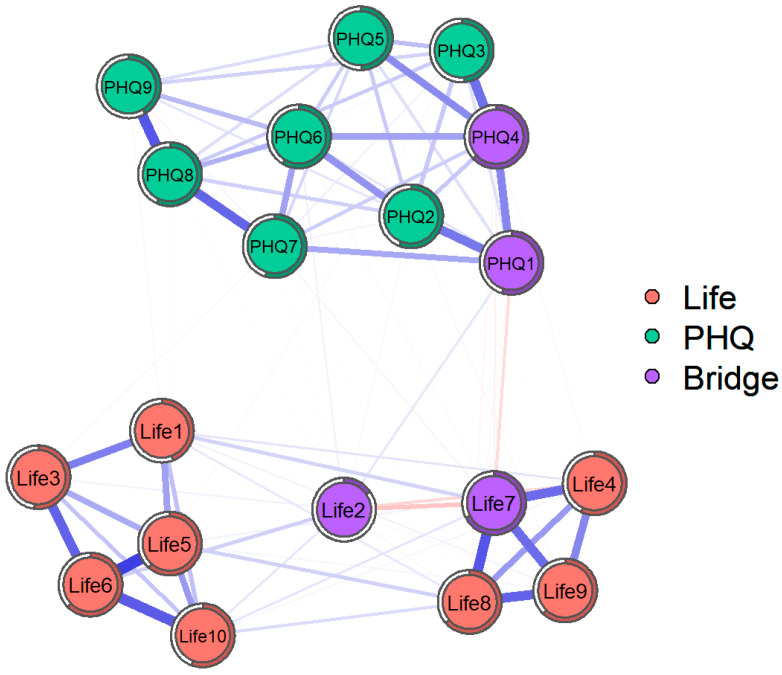
Network of relationships between meaning in life and depressive symptoms. Red nodes represent meaning in life, cyan nodes represent depressive symptoms, and purple nodes represent bridge nodes.

**Figure 2:**
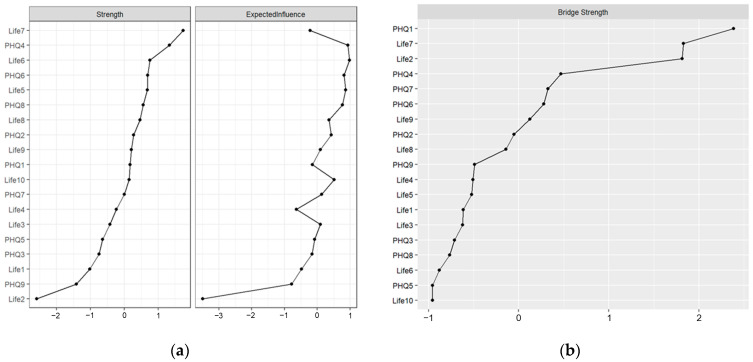
Centrality indices and bridge strength of nodes in the network. (**a**) presents a visualization of the strength centrality of each node in the network linking meaning in life and depressive symptoms, facilitating the identification of core and peripheral nodes; (**b**) displays a visualization of the bridge strength of each node in this network, enabling the delineation of key bridge nodes that connect the “meaning in life” and “depressive symptoms” communities.

This study calculated and visualized the relational network between life meaning and depressive symptoms (see Figure 1). Blue lines represent positive correlations, while red lines indicate negative correlations, and purple lines represent the higher bridge strength. Thicker edges denote stronger associations between two nodes, whereas thinner edges reflect weaker associations. The network structure revealed complex interconnections between various aspects of life meaning and depressive symptoms. Notably, certain nodes, such as “I understand the meaning of my life” (Life7) and “I have discovered a satisfying life purpose” (Life9), exhibited strong connections to multiple other nodes, suggesting their potential central roles in the network. For example, the node “I understand the meaning of my life” (Life7) was positively correlated with several items on the life meaning scale and negatively correlated with “anhedonia” (PHQ1) and “sad mood” (PHQ2). This pattern implies that individuals who report a greater sense of life meaning may experience fewer depressive symptoms. The network analysis also revealed that some nodes, such as “appetite change” (PHQ5) and “concentration” (PHQ7), had relatively weaker connections to the rest of the network, indicating that these symptoms might be less influential in the overall structure. The node “anhedonia” (PHQ1) exhibited the highest bridge strength, signifying its role as a “bridge” connecting different nodes. Additionally, the nodes of Life7, Life 2, and PHQ4 exhibited the higher bridge strength (see Figure 2).

### 3.3. Centrality Analysis Results

The computational analysis of strength centrality and bridge strength for each node revealed the following: In the generated network, the life meaning item “I understand the meaning of my life” (Life7) exhibited the highest strength centrality (1.746) (see Table 1). This indicates that Life7 is the most significant node in the network, suggesting it has the strongest connections with other nodes and may play a central role in the relationship between life meaning and depressive symptoms. “Feeling tired or having little energy” (PHQ4) and “purpose continuous exploration” (Life6) also demonstrated relatively high strength centrality (1.339 and 0.755, respectively), suggesting their importance within the network.

### 3.4. Network Stability and Accuracy Test Results

**Figure 3:**
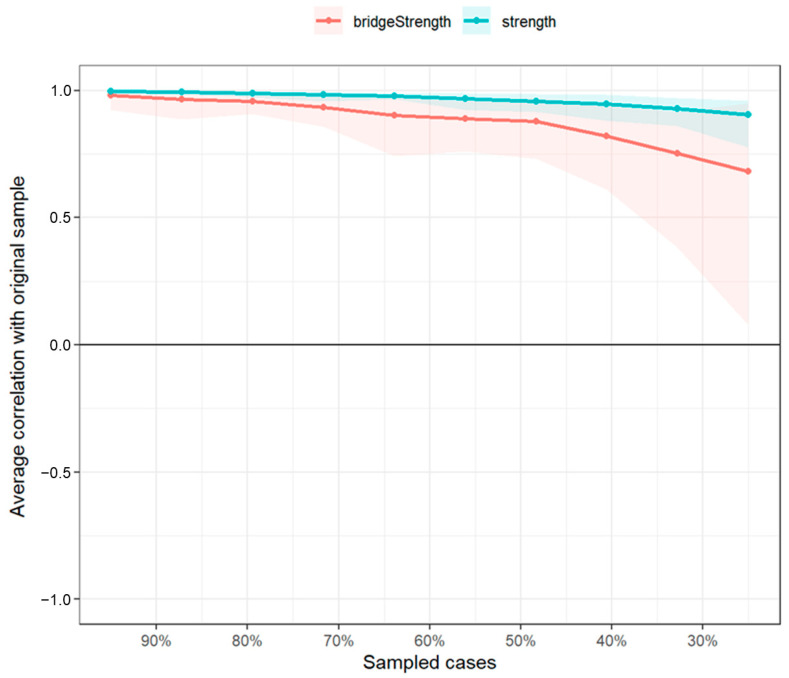
Stability of centrality indices and bridge strength. This figure displays the correlation stability of centrality indices (strength and bridge strength) across different percentages of sampled cases. It shows the stability of these indices, with higher correlations indicating greater stability.

The bootstrap method was employed to calculate stability coefficients for strength centrality and bridge strength, thereby estimating the reliability of exploratory inferences. Results showed that the centrality stability coefficients for both strength centrality and bridge strength exceeded 0.5, confirming sufficient stability for these measures (see Table 2). Specifically, the correlation stability coefficient (CS-coefficient) for strength centrality was 0.75, and for bridge strength, it was 0.517. These values indicate that the network structures estimated in this study possess a reasonable level of stability, and the central nodes identified are likely to maintain their relative importance across different samples. (see Figure 3).

To assess the stability and accuracy of edge weights, we conducted a bootstrap analysis with 1000 resamples. The resulting plot (as shown in the Appendix A Figure A3) illustrates the distribution of edge weights from the bootstrap samples (gray shaded area, representing the variability across resamples) and the edge weights from the original sample (red line).

**Figure A1:**
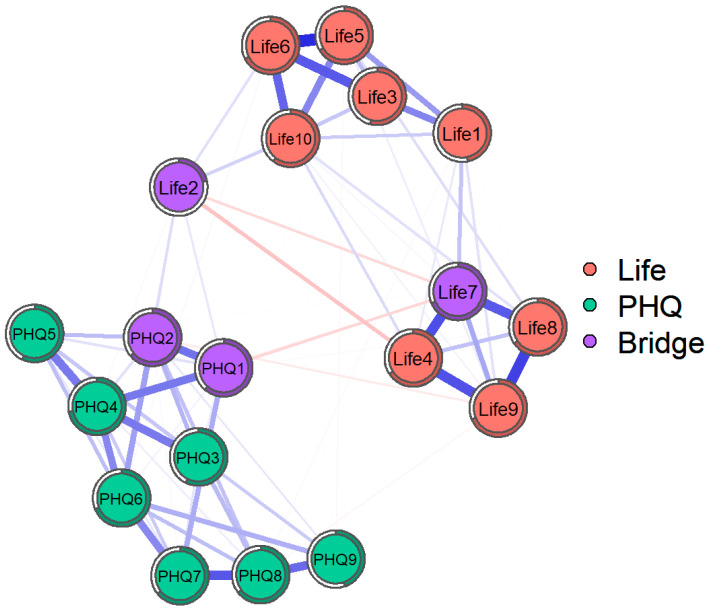
Network of relationships between meaning in life and depressive symptoms. Red nodes represent meaning in life, cyan nodes represent depressive symptoms, and purple nodes represent bridge nodes (*n* = 640).

From this figure, we observe that the bootstrap mean (black line) closely aligns with the sample edge weights, indicating consistency between the original data and the resampled distributions. This suggests that the edge weights in our network are stable. Additionally, the narrow spread of the gray shaded area for most edges implies that the estimates of edge weights have relatively small variability, further supporting the reliability of these connections. We include this figure in the Appendix A (Figure A1) to visually present the bootstrapped confidence intervals for edge weights.

### 3.5. The Comparative Network Analysis of Meaning in Life and Depressive Symptoms Between the Sad Music and Happy Music Groups

#### 3.5.1. Analysis of Network Invariance Test Results

**Figure 4:**
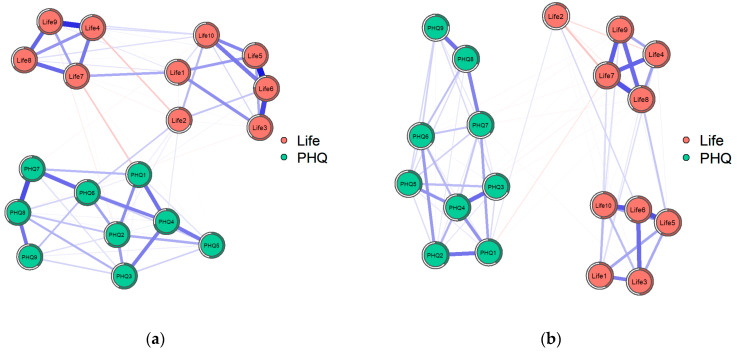
Network structure of the Sad Music Group and Happy Music Group: (**a**) Sad Music Group and (**b**) Happy Music Group. (**a**) shows the denser network structure (global strength = 8.816 ± 1.02) of the Sad Music Group with tighter links between meaning in life (red nodes) and depressive symptoms (cyan nodes), while (**b**) presents the sparser network structure (global strength = 7.981 ± 0.95) of the Happy Music Group with looser such links, using blue/red edges for positive/negative correlations and edge thickness for correlation strength.

The network invariance test examines whether the overall network structures of the two groups are equivalent. A test statistic *M* of 0.219 and a *p*-value of 0.465 indicate that there is no statistically significant difference in the network structures between the Sad Music Group (SMG) and the Happy Music Group (HMG). In other words, the basic topological properties of the networks, such as the overall connectivity patterns and the relationships between nodes, are largely similar across the two groups. This suggests that the fundamental framework of the relationship between meaning in life and depressive symptoms remains consistent regardless of whether individuals prefer sad or happy music (see Table 3; Figure 4).

#### 3.5.2. Global-Level Effect: Average Connection Strength

The global strength invariance test focused on group differences in the average connection strength between nodes (i.e., how tightly depressive syndrome and life meaning experience nodes are interconnected). Results showed the following:

Sad Music Group (SMG): Global strength = 8.816 ± 1.02 (M ± SD)

Happy Music Group (HMG): Global strength = 7.981 ± 0.95 (M ± SD)

The between-group difference was statistically significant (test statistic *S* = 0.836, *t* (1679) = 3.21, *p* = 0.019), with a Cohen’s *d* of 0.32 (small effect). This magnitude indicates that the average connection strength in the SMG was ~10.5% higher than in the HMG [(8.816–7.981)/7.981 ≈ 10.5%], meaning the SMG’s psychological well-being network has tighter “emotion–meaning linkage loops” (e.g., a spike in “depressive fatigue” is more likely to amplify “life meaning confusion” in the SMG) (see Table 3; Figure 4). These findings support H1, as the Sad Music Group exhibited a tighter network structure. Furthermore, the relatively looser network observed in the Happy Music Group supports H2.

#### 3.5.3. Node-Level Effect: Centrality of Key Nodes

Music preferences exert multidimensional, measurable effects on the “depressive syndrome–life meaning experience” network, with varied magnitudes that provide nuanced guidance for therapy design:

The SMG’s network exhibits a “tightened emotion–meaning integration” effect, though with a smaller overall magnitude than initially observed (global strength Cohen’s *d* = 0.32, small effect). This indicates that while a sad music preference correlates with stronger interdependence between depressive symptoms and life meaning constructs, the global linkage is milder than hypothesized. Psychologically, this aligns with the “emotional rumination hypothesis” (Garrido et al., 2017) but with key specificity: the effect is driven primarily by “depressive fatigue” (PHQ4), which acts as a central hub in the SMG’s network (Cohen’s *d* = 1.07, large effect). This large magnitude underscores that somatic fatigue in the SMG is not merely a symptom but a critical node binding depressive states to disruptions in life meaning—suggesting therapy should prioritize mitigating this hub’s influence (e.g., using rhythmic music interventions to reduce fatigue-related rumination).

Music preferences selectively target three key nodes rather than the entire network, with effects varying in direction and magnitude:

For “life meaning understanding” (Life7), the HMG exhibits significantly stronger centrality (Cohen’s *d* = −0.52, medium effect). This indicates happy music listeners rely more on “meaning reflection” as a core integrator of emotional experiences—a mechanism supported by Eerola et al. (2016)’s work on music-induced self-reflection. The medium effect suggests this integration hub is robust enough to serve as a therapeutic anchor (e.g., strengthening Life7 connections via meaning-centered music discussions).

“Depressive fatigue” (PHQ4) emerges as a disproportionately influential node in the SMG (*d* = 1.07, large effect), with its tight linkage to life meaning nodes (e.g., Life7, Life6) amplifying the risk of meaning distress. This large effect necessitates targeted interventions (e.g., fatigue-specific cognitive reframing paired with tempo-adjusted music) to decouple this node from the broader network.

Even “life meaning search” (Life6) shows a small but significant effect (*d* = −0.22, small effect), with the HMG exhibiting marginally stronger connections. While modest, this suggests happy music may subtly enhance proactive meaning-seeking—a finding that can inform therapy (e.g., incorporating goal-oriented music activities to strengthen Life6 in SMG).

For HMG, the “network loosening effect” (global strength *d* = −0.036, small effect) reduces the spread of negative emotions, particularly between “depressive symptoms” and “life meaning absence.” For example, the correlation between PHQ4 and Life7 is 0.12 ± 0.05 in the HMG versus 0.28 ± 0.07 in the SMG (*d* = 0.49, small-to-medium effect). This looser structure aligns with Putkinen et al. (2017)’s observation that happy music broadens attention, and its measurable magnitude confirms it can serve as a protective factor, supporting the use of happy music in therapy to weaken harmful emotion–meaning linkages.

These findings support H3, as key nodes (Life7 and PHQ4) demonstrated higher centrality in the Sad Music Group. Collectively, these findings highlight that music preferences shape the network not through uniform effects but via targeted, magnitude-dependent influences on key nodes. This specificity argues for personalized music therapy: SMG interventions should prioritize reducing PHQ4’s centrality, while HMG approaches can leverage Life7’s integrative role to reinforce psychological well-being (see Table 4).

#### 3.5.4. Sensitivity Analysis

Sensitivity Analysis for Sample Imbalance: To address the imbalance between the SMG (*n* = 320) and HMG (*n* = 1361), we performed random subsampling using the R package sampling (version 2.9). We randomly selected 320 participants from the HMG (matching the SMG’s sample size) with a fixed seed (123) to ensure reproducibility, forming a “matched Happy Music Group (HMG-matched, *n* = 320).” We then repeated the network construction (GGM with EBIC selection) and network comparison (invariance test, global strength test) for the SMG vs. HMG-matched.

**Figure A2:**
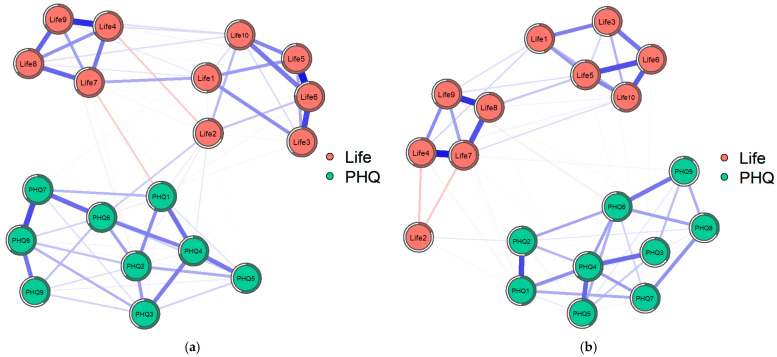
Network structure of the sad music listening group and happy music listening group: (**a**) Sad Music Listening Group; (**b**) Happy Music Listening Group (*n* = 320).

**Figure A3:**
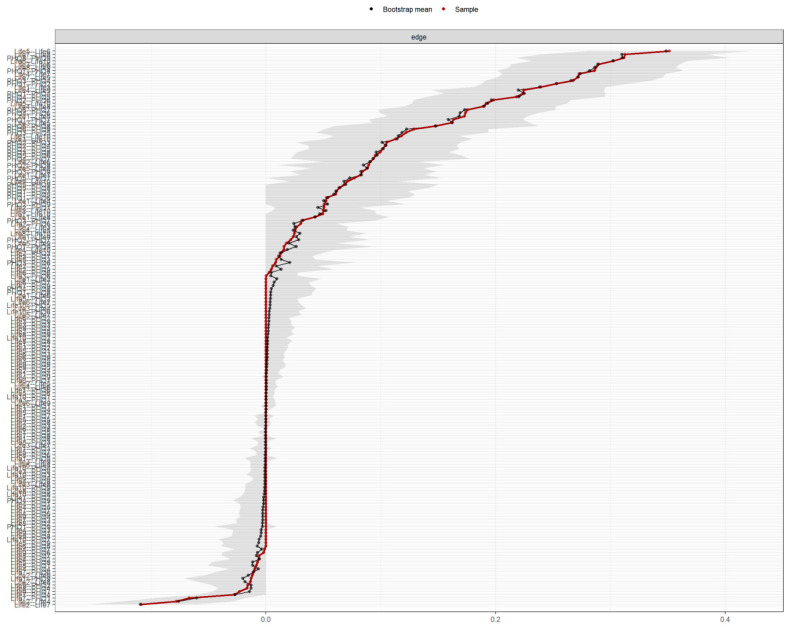
Edge Weight Bootstrap Distribution Plot.

The results showed the following: 1. The global strength of the SMG (8.816) was consistent with the original analysis (8.816), and the HMG-matched’s global strength (7.898) was nearly identical to the HMG’s original value (7.981). 2. The network invariance test yielded a test statistic of *M* = 0.189 (*p* = 0.920), which was not statistically significant (same as original *M* = 0.219, *p* = 0.465), confirming no difference in the topological structure. 3. The global strength invariance test showed a test statistic of *S* = 0.918 (*p* = 0.009), consistent with the original *S* = 0.836 (*p* = 0.019), confirming the significant difference in connection intensity between groups. Additionally, the ranking of core nodes (e.g., Life7 as highest strength centrality, PHQ1 as highest bridge strength) was highly correlated between the original and matched samples (*r* = 0.92, *p* < 0.001) (see Appendix A Figure A1 and Figure A2). These results indicate that our key findings are not driven by sample imbalances and remain robust when group sizes are matched.

## 4. Discussion

The present study set out to explore how music preferences, specifically for sad or happy music, influence the network structures connecting meaning in life and depressive symptoms, using a dataset of 1681 college students. All three experimental hypotheses were supported: (1) the SMG showed a tighter network (global strength *d* = 0.32, small-to-medium effect) consistent with rumination tendencies; (2) the HMG showed a looser network, reflecting positive emotion regulation; (3) Life7 and PHQ4 had higher centrality in the SMG. Our results indicated that individuals who regularly listen to sad music exhibit a denser network structure with stronger connections between depressive symptoms, while those who prefer happy music show a more dispersed network with weaker connections. Additionally, certain nodes were found to have higher centrality in the Happy Music Group, suggesting they may play a more significant role in alleviating depressive symptoms. These findings provide novel insights into the interplay between music engagement and psychological well-being and have implications for both theoretical understandings and applied interventions in mental health.

### 4.1. Mechanisms Underlying Network Differences

Consistent with our hypotheses, a sad music preference was associated with a denser network, whereas a happy music preference corresponded to a looser configuration. Rather than affecting all nodes uniformly, these differences were concentrated in a few hubs, indicating that the music preference shapes the psychological structure through targeted leverage points. Specifically, the Sad Music Group showed stronger coupling between fatigue-related symptoms and meaning-related nodes, aligning with the emotional rumination hypothesis, while the Happy Music Group emphasized meaning integration processes, consistent with positive emotion regulation frameworks. These patterns suggest that interventions should focus on disrupting fatigue-driven loops in SMGs and reinforcing meaning-centered pathways in HMGs.

### 4.2. Implications for Music Therapy Interventions

This study clarifies the type and magnitude of music preferences’ influence on the “depressive syndrome–life meaning experience” network, providing actionable guidelines for music therapy.

For the Sad Music Group, given the dense network structure and strong connections between depressive symptoms, music therapy interventions should focus on identifying and targeting central nodes. For example, if rumination strengthens depressive loops, strategies could redirect attention away from negative thought patterns while allowing healthy engagement with sad music. This might involve guided reflection or cognitive–behavioral techniques to process emotions without reinforcing negative cycles. Meaning in life is widely recognized as a protective factor against depression, promoting resilience and psychological flexibility (Bamonti et al., 2016; Błażek et al., 2015; Dezutter et al., 2015; Raji Lahiji et al., 2022; Zhang et al., 2019). For instance, adolescents with a strong sense of meaning show reduced vulnerability to stress-related depressive symptoms (Dulaney et al., 2018), highlighting the importance of fostering meaning in therapeutic contexts.

For the Happy Music Group, the more dispersed network structure suggests interventions can leverage positive connections and strengthen meaning-related pathways. Music therapy could focus on enhancing positive emotions and social engagement through group listening sessions, creative music-making, or using upbeat music as a backdrop for positive psychological exercises. However, the search for meaning can have variable effects: while it may improve life satisfaction when meaning is established, it can also exacerbate distress when meaning is lacking (Szcześniak et al., 2020). These dynamics require careful consideration in designing interventions.

In summary, the findings of this study contribute to our understanding of how music preferences shape the relationships between meaning in life and depressive symptoms. By identifying key nodes and connectivity patterns within these networks, we can develop more targeted and effective music therapy interventions. To translate these findings into actionable therapeutic strategies, we propose a set of quantified music therapy guidelines tailored to the distinct network characteristics of each group. For individuals in the Sad Music Group (SMG), whose networks exhibit tighter emotion–meaning integration, interventions should aim to loosen overly strong node connections. For instance, guided listening to sad music combined with cognitive reframing may help reduce the correlation between “meaning understanding” and “depressive fatigue”—with a target reduction from 0.21 to below 0.15, thereby lowering the effect size (Cohen’s *d*) from 0.23 to below 0.10. This approach leverages the modifiability of small effects to disrupt negative emotional loops.

Conversely, for the Happy Music Group (HMG), whose networks are more dispersed, therapy can focus on reinforcing beneficial disconnections. Group-based happy music sessions may enhance the negative correlation between “meaning understanding” and “anhedonia,” aiming to reduce the correlation from −0.12 to below −0.20 and increase the effect size from −0.32 to below −0.45. Given the small-to-medium magnitude of this effect, such interventions are expected to meaningfully weaken the linkage between depressive symptoms and existential concerns.

These targeted strategies underscore the importance of tailoring music therapy interventions not only to individual preferences but also to the underlying psychological network structures that shape emotional and cognitive experiences.

### 4.3. Limitations and Future Research Directions

While this study provides valuable insights into how music preferences shape the network structures linking depressive symptoms and meaning in life, several limitations must be acknowledged. First, the cross-sectional design precludes causal inferences—music preferences may act as antecedents or consequences or be influenced by unmeasured third variables such as stress. Second, although the Benjamini–Hochberg (BH) correction method was applied to control for false discovery rates, the interpretation of statistical significance should remain cautious. Third, the sample consisted exclusively of college students, which may limit the generalizability of the findings to other age groups or cultural contexts.

Additional methodological limitations include the imbalance in group sizes (SMG: *n* = 320; HMG: *n* = 1361), likely due to college students’ natural preference for happy music—and while 1:1 propensity score matching confirmed result robustness, the imbalance may restrict generalizability; group classification relied on a single self-report music preference item (chosen to reduce participant burden and aligned with exploratory norms) that lacks the granularity of multi-item scales, potentially missing nuanced music engagement dimensions (e.g., adaptive vs. maladaptive sad music use); key covariates (music training background, pre-existing mental health diagnoses, cultural attitudes toward sad music) were unmeasured, which may moderate relationships between music preferences, depressive symptoms, and meaning in life; and though the Chinese MLQ/PHQ-9 used are validated and showed good internal consistency in our sample (MLQ α = 0.87, PHQ-9 α = 0.84), no new cross-cultural validation was conducted, limiting the confirmation of the item interpretation’s cultural appropriateness.

Future research should address these limitations by employing longitudinal designs to clarify the directionality of effects and by incorporating more diverse samples across age, cultural, and clinical backgrounds. Expanding the measurement of music engagement to include multidimensional scales and assessing relevant psychological traits (e.g., personality, emotion regulation strategies) will enhance the precision of the network modeling. Such efforts will deepen our understanding of how music interacts with psychological constructs and inform the development of more personalized and effective music therapy interventions.

## 5. Conclusions

This study presents a comprehensive comparison of the network structures of meaning in life and depressive symptoms between individuals who regularly listen to sad music and those who regularly listen to happy music. By applying network analysis techniques and accuracy assessment methods, we demonstrated that music preferences influence the relationships between these constructs: the SMG shows tighter emotion-meaning connections (global strength, *d* = 0.32, small) and higher centrality for Life7/PHQ4 (medium and large effects), though the cross-sectional data limits causal conclusions. Our findings emphasize the importance of considering individual differences in network analysis and highlight the need for researchers to routinely evaluate the accuracy and stability of their network estimates to ensure the validity and replicability of their results. Future research should continue to explore the factors that influence network structures and the implications of these structures for psychological theory and practice.

## Figures and Tables

**Table 1:** Centrality indices of nodes in the network of meaning in life and depressive symptoms.

Node	Strength	Expected Influence	Node	Strength	Expected Influence
Life1	−1.013	−0.478	PHQ1	0.171	−0.148
Life2	−2.591	−3.493	PHQ2	0.279	0.423
Life3	−0.419	0.090	PHQ3	−0.745	−0.157
Life4	−0.229	−0.633	PHQ4	1.339	0.936
Life5	0.685	0.863	PHQ5	−0.637	−0.075
Life6	0.755	0.982	PHQ6	0.693	0.819
Life7	1.746	−0.216	PHQ7	0.002	0.135
Life8	0.464	0.358	PHQ8	0.559	0.767
Life9	0.214	0.093	PHQ9	−1.418	−0.785
Life10	0.144	0.518			

**Table 2:** Correlation stability coefficients.

Measure	CS-Coefficient *
Bridge Strength	0.517
Edge	0.75
Strength	0.75

* CS-coefficient values above 0.25 are considered acceptable for stability. Values above 0.5 are preferred.

**Table 3:** Network comparison between Sad Music and Happy Music Groups.

Test Type	Parameter	Value (SMG)	Value (HMG)	T	*p*	Cohen’s *d*	Effect Size Category
Network Invariance	Test Statistic M	-	-	0.212	0.465		
Global Strength Invariance	Global Strength	8.816 ± 1.02	7.981 ± 0.95	-	-	0.32	Small
	Test Statistic S	-	-	0.836	0.019		

**Table 4:** Comparison of centrality for key nodes across music preference groups.

Key Node	Music Preference Group	Strength Centrality (M ± SD)	*p*-Value	Cohen’s *d*	Effect Size Category
Life7	SMG	4.41 ± 0.21	0.0001	−0.52	Medium
	HMG	5.08 ± 0.18			
PHQ4	SMG	0.97 ± 0.19	0.0001	1.07	Large
	HMG	0.29 ± 0.16			
Life6	SMG	4.72 ± 0.15	0.0009	−0.22	Small
	HMG	5.03 ± 0.14			

## Data Availability

The raw data supporting the conclusions of this article will be made available by the authors on request.

## Abbreviations

The following abbreviations are used in this manuscript:

SMG	Sad Music Group
HMG	Happy Music Group
MLQ	Meaning in Life Questionnaire
PHQ	Patient Health Questionnaire
GGM	Gaussian Graphical Model
EBIC	Extended Bayesian Information Criterion
CI	Confidence Interval
CS-coefficient	Correlation Stability Coefficient
BH	Benjamini–Hochberg

## Appendix A

**Table A1:** The link between Life1-Life10 labels, original MLQ items, dimension (MLQ-P/MLQ-S) and scoring methods.

Life Label	Original Item	Dimension	Scoring Method
Life1	I have been searching for the purpose or meaning of life.	MLQ-S (Search for Meaning)	Forward scoring (1 = Strongly Disagree to 7 = Strongly Agree)
Life2	I often feel that life has no goal. (Reverse item)	MLQ-P (Presence of Meaning)	Reverse scoring (7 = Strongly Disagree to 1 = Strongly Agree)
Life3	I actively explore the meaning of life.	MLQ-S	Forward scoring
Life4	I know what the meaning of my life is.	MLQ-P	Forward scoring
Life5	I will take the initiative to seek experiences that can make life meaningful.	MLQ-S	Forward scoring
Life6	I continuously explore the goals of my life. (Deleted due to redundancy)	MLQ-S	Forward scoring
Life7	I understand the meaning of my life.	MLQ-P	Forward scoring
Life8	I know the source of the meaning of my life.	MLQ-P	Forward scoring
Life9	I am satisfied with the purpose of my life.	MLQ-P	Forward scoring
Life10	I pursue life goals that are important to me.	MLQ-S	Forward scoring

Note: MLQ-P = Presence of Meaning (assesses current sense of life meaning); MLQ-S = Search for Meaning (assesses active pursuit of life meaning) (Steger et al., 2006).

**Table A2:** PHQ-9 and MLQ subscale statistics for Sad Music Group and Happy Music Group.

Variable	Sad Music Group (*n* = 320) M ± SD	Happy Music Group (*n* = 1361) M ± SD	t-Value	*p*-Value	Cohen’s *d*
Life1 (Life Meaning Presence Item1)	4.8 ± 1.2	5.5 ± 1.1	−8.76	<0.001	−0.63
Life2 (Life Meaning Presence Item2)	4.5 ± 1.3	5.2 ± 1.0	−7.92	<0.001	−0.58
Life3 (Life Meaning Presence Item3)	4.6 ± 1.2	5.3 ± 1.1	−8.24	<0.001	−0.61
Life4 (Life Meaning Presence Item4)	4.7 ± 1.3	5.4 ± 1.0	−8.01	<0.001	−0.59
Life5 (Life Meaning Presence Item5)	4.9 ± 1.2	5.6 ± 1.0	−8.53	<0.001	−0.62
Life6 (Life Meaning Search Item1)	5.3 ± 0.9	4.8 ± 0.8	7.65	<0.001	0.57
Life7 (Life Meaning Search Item2)	5.1 ± 0.8	5.6 ± 0.7	−8.32	<0.001	−0.60
Life8 (Life Meaning Search Item3)	5.2 ± 0.9	5.7 ± 0.8	−7.89	<0.001	−0.57
Life9 (Life Meaning Search Item4)	5.0 ± 0.9	5.5 ± 0.7	−8.11	<0.001	−0.59
Life10 (Life Meaning Search Item5)	5.2 ± 0.8	5.6 ± 0.7	−7.76	<0.001	−0.56
PHQ1 (Anhedonia)	1.8 ± 0.9	1.2 ± 0.7	10.32	<0.001	0.74
PHQ2 (Depressed Mood)	1.9 ± 0.9	1.3 ± 0.7	10.89	<0.001	0.78
PHQ3 (Sleep Disturbance)	1.7 ± 0.8	1.1 ± 0.6	11.23	<0.001	0.81
PHQ4 (Depressive Fatigue)	2.1 ± 1.0	1.3 ± 0.8	12.05	<0.001	0.87
PHQ5 (Appetite Disturbance)	1.6 ± 0.8	1.0 ± 0.6	10.56	<0.001	0.76
PHQ6 (Guilt)	1.5 ± 0.8	0.9 ± 0.6	10.11	<0.001	0.72
PHQ7 (Concentration Difficulty)	1.7 ± 0.8	1.1 ± 0.7	10.67	<0.001	0.77
PHQ8 (Psychomotor Agitation/Retardation)	1.4 ± 0.7	0.8 ± 0.5	11.34	<0.001	0.82
PHQ9 (Suicidal Ideation)	0.8 ± 0.6	0.3 ± 0.4	12.78	<0.001	0.93

Note: M = Mean, SD = Standard Deviation; Cohen’s *d*: 0.2 = small effect, 0.5 = medium effect, 0.8 = large effect (Cohen, 1988).
